# Ginkgetin: A Promising Multitarget Agent for Diverse Diseases

**DOI:** 10.3390/biom16040488

**Published:** 2026-03-24

**Authors:** Zhitong Sun, Zhijian Rao, Yibing Lu, Xingwen Zheng, Lifang Zheng

**Affiliations:** 1 College of Physical Education, Shanghai University, Shanghai 200444, China; 1287635496@shu.edu.cn (Z.S.); luyibing0513@shu.edu.cn (Y.L.); zxw2003@shu.edu.cn (X.Z.); 2 College of Physical Education, Shanghai Normal University, Shanghai 200238, China; raoz19@shnu.edu.cn; 3 Exercise Biological Center, China Institute of Sport Science, Beijing 100061, China

**Keywords:** ginkgetin, biflavonoid, multitarget pharmacology, molecular mechanisms, therapeutic potential, preclinical studies

## Abstract

Ginkgetin (GK) is a naturally occurring biflavonoid predominantly isolated from *Ginkgo biloba* and has attracted increasing attention because of its broad pharmacological activities. Structurally, GK belongs to the 3′-8″-linked biflavone subclass, which distinguishes it from other biflavonoids like amentoflavone (the parent compound of this subclass) and its monomeric counterparts such as apigenin. This unique C-C linked dimeric architecture confers distinct molecular planarity and lipophilicity, contributing to its enhanced membrane permeability and multitarget engagement capabilities. GK has been shown to exert pleiotropic biological effects in preclinical studies, including anti-inflammatory, antioxidant, antifibrotic, anticancer, neuroprotective, cardioprotective, metabolic regulatory and antibacterial activities. Mechanistically, preclinical evidence indicates that GK functions as a multitarget modulator of key signaling pathways involved in oxidative stress, inflammation, cell death and tissue remodeling, such as nuclear factor erythroid 2–related factor 2/heme oxygenase-1 (Nrf2/HO-1), nuclear factor kappa-B(NF-κB), Janus kinase/signal transducer and activator of transcription(JAK/STAT), mitogen-activated protein kinases(MAPKs), AMP-activated protein kinase/mechanistic target of rapamycin(AMPK/mTOR), phosphoinositide 3-kinase/protein kinase B(PI3K/Akt) and cyclic GMP-AMP synthase–stimulator of interferon genes(cGAS–STING). Notably, GK has been observed to display context-dependent regulation of cell fate decisions, including apoptosis, autophagy and ferroptosis, thereby enabling the selective elimination of pathological cells while preserving normal tissue function. Preclinical studies further demonstrate that GK exhibits therapeutic potential across diverse disease systems, including cancer, metabolic disorders, cardiovascular diseases, neurological disorders and musculoskeletal diseases. In addition, emerging evidence highlights its antibacterial and antivirulence properties through the inhibition of biofilm formation and quorum sensing. It is crucial to note, however, that this promising profile is predominantly derived from preclinical studies, and clinical evidence in humans remains to be established. Despite these promising findings, the clinical translation of GK remains limited by challenges related to pharmacokinetics, bioavailability and druggability. This review systematically summarizes the chemical characteristics, pharmacological activities and molecular mechanisms of GK, with an emphasis on its multitarget actions and therapeutic potential across disease systems, and discusses current limitations and future perspectives to facilitate the rational development of GK-based interventions.

## 1. Introduction

The increasing global burden of chronic and complex diseases, including cancer, metabolic disorders, cardiovascular diseases, neurodegenerative diseases and degenerative musculoskeletal conditions, poses a major challenge to current healthcare systems [1,2]. These diseases are typically characterized by multifactorial pathogenesis involving oxidative stress, chronic inflammation, dysregulated cell death, metabolic imbalance and immune dysfunction [3,4]. Despite significant advances in modern medicine, conventional single-target therapies often exhibit limited efficacy and are frequently associated with adverse effects, highlighting the urgent need for novel therapeutic strategies with improved efficacy and safety profiles [5].

Natural products have long served as a critical source of therapeutic agents owing to their structural diversity and broad biological activities [6]. In particular, polyphenolic compounds have attracted increasing attention because of their ability to simultaneously modulate multiple signaling pathways, thereby exerting pleiotropic protective effects across diverse disease contexts [6,7]. Such multitarget characteristics are especially advantageous for the treatment of complex diseases driven by interconnected molecular networks [8].

Ginkgetin (GK), a naturally occurring biflavonoid initially isolated from *Ginkgo biloba*, represents one such promising multitarget compound [9]. Accumulating preclinical evidence demonstrates that GK exhibits a wide spectrum of biological activities, including anti-inflammatory, antioxidant, antifibrotic, anticancer, neuroprotective, antibacterial and anti-aging effects [10,11,12,13,14,15,16,17,18,19]. Mechanistically, GK has been shown to regulate a variety of key signaling pathways, such as Nrf2/HO-1, NF-κB, JAK/STAT, MAPKs, AMPK/mTOR and PI3K/Akt, which are critically involved in disease initiation and progression [10,15,17,18,19]. Importantly, GK displays context-dependent regulatory effects on cell fate decisions, including apoptosis, autophagy and ferroptosis, enabling selective elimination of pathological cells while protecting normal tissues under stress conditions [10,15,19,20]. In addition, preclinical studies suggest that GK can synergize with existing therapeutic agents, enhance treatment efficacy and potentially overcome drug resistance, particularly in cancer therapy [20].

While flavonoids like quercetin have been extensively studied, GK presents a distinct and compelling case for a focused, in-depth review at this time, based on its unique chemical attributes and emerging biological profile. Unlike common monomeric flavonoids, GK’s distinctive C-C linked biflavonoid scaffold confers superior molecular planarity and lipophilicity, which likely underpins its enhanced membrane permeability and differential engagement with key therapeutic targets [21]. More importantly, GK demonstrates a unique and context-sensitive therapeutic window that is not readily replicated by other flavonoids. This is evidenced by its dual capability to simultaneously suppress pathogenic drivers (e.g., via NF-κB inhibition) while potently activating cytoprotective pathways (e.g., via Nrf2/HO-1 induction), a balanced multitarget action that may translate to an improved efficacy-safety ratio in complex diseases [22]. Furthermore, recent years have witnessed a surge of high-impact studies revealing GK’s novel and potent roles in modulating cutting-edge pathways such as cGAS-STING in sterile inflammation and immunometabolism [23], as well as its precise regulation of emerging cell death modalities like ferroptosis [15]. These recent discoveries, scattered across diverse disease models, have created a critical knowledge gap: the lack of an integrated analysis that connects GK’s unique structural identity to its pleiotropic mechanisms and its emergent therapeutic niche. This review aims to fill this gap.

Despite the growing interest in GK, a comprehensive and mechanism-oriented synthesis of its pharmacological actions across disease systems remains lacking. Therefore, the present review systematically summarizes the chemical characteristics, natural sources and pharmacological profile of GK, critically analyzes its underlying molecular mechanisms and discusses its therapeutic potential across diverse disease systems. A critical perspective is maintained throughout this review, acknowledging that while the preclinical data are compelling, the translational journey of GK is at an early stage, with a clear need for rigorous clinical validation to ascertain its efficacy and safety in humans. Furthermore, current limitations and future perspectives are highlighted to facilitate the rational development and clinical translation of GK-based interventions.

## 2. Chemical Characteristics, Natural Sources and Pharmacological Profile of Ginkgetin

### 2.1. Chemical Structure and Physicochemical Properties, and Structure–Activity Relationships

**Figure 1:**
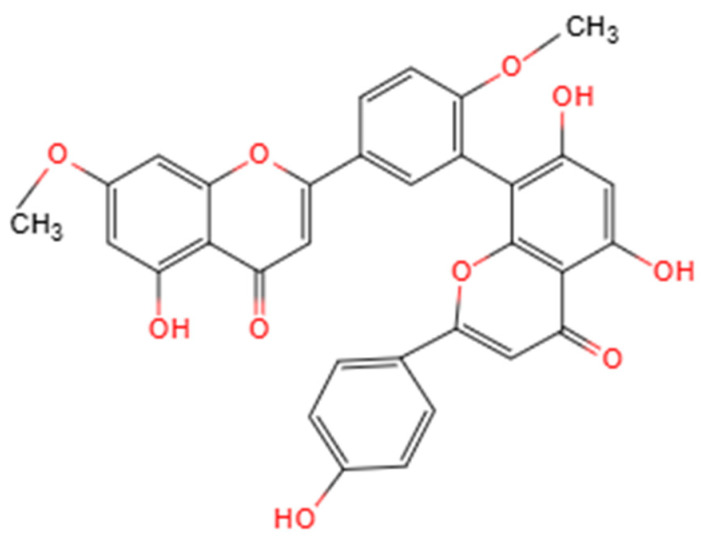
Structure of Ginkgetin. Created by the authors.

GK is a naturally occurring biflavonoid composed of two apigenin units linked through a C8–C8′ carbon-carbon (C-C) bond, forming a distinctive dimeric structure that places it within the 3′-8″-linked biflavone subclass [21,24] (Figure 1). This unique molecular architecture, characterized by the biphenyl linkage, differentiates GK from monomeric flavonoids and is a fundamental determinant of its enhanced chemical stability and its ability to interact with multiple molecular targets [25]. The presence and specific positioning of methoxy (-OCH_3_) and hydroxyl (-OH) groups on the flavone scaffolds are critical for its biological activity, endowing GK with pronounced redox-regulating capacity and protein-binding potential [9]. From a physicochemical perspective, GK exhibits relatively high lipophilicity and poor aqueous solubility, characteristics that may substantially influence its absorption, distribution and bioavailability in vivo [25].

A deeper analysis of the structure–activity relationship (SAR) provides mechanistic insights into GK’s pleiotropic effects. The biological profile of GK arises from the synergistic interplay between its functional group chemistry and its unique dimeric scaffold. The specific location of hydroxyl and methoxy groups dictates target affinity and functional selectivity [26]. For antioxidant and cytoprotective activities linked to the Nrf2 pathway, the presence of a catechol moiety (ortho-dihydroxy groups) on the B-ring is crucial. This structural motif facilitates electron transfer and metal chelation, which are key for interacting with the reactive cysteine sensors of *Keap1*, thereby stabilizing Nrf2 and promoting the transcription of downstream antioxidant genes [22]. Concurrently, the methoxy substitutions modulate the compound’s overall electronic character and enhance its lipophilicity, which is vital for cellular uptake and membrane association. Regarding the modulation of the cGAS-STING pathway, emerging molecular docking evidence suggests that GK can bind directly to the STING protein [23]. This interaction is proposed to involve hydrogen bonding via specific hydroxyl groups and extensive hydrophobic contacts. The methoxy groups, by increasing the molecule’s lipophilicity and planar aromatic surface area, likely optimize π-π stacking interactions with key hydrophobic residues (e.g., tryptophan, phenylalanine) within the STING binding pocket, thereby enhancing binding affinity and inhibitory potency.

The C-C biphenyl linkage is not merely a passive scaffold; it is integral and necessary for GK’s distinctive bioactivity. This linkage imposes a specific, relatively constrained dihedral (“twist”) angle between the two apigenin units, resulting in a semi-rigid, extended planar conformation [21]. This unique three-dimensional shape is critical for molecular recognition, as it presents a large, continuous hydrophobic surface that maximizes van der Waals contacts and achieves optimal shape complementarity with the binding pockets of diverse target proteins, such as kinases and transcription factors [25]. Furthermore, the dimeric structure effectively doubles the pharmacophore, enabling potential bidentate or cooperative binding to a single target or simultaneous engagement with proximal sites on protein complexes [27]. This feature can lead to significantly higher binding affinity and specificity compared to monomeric flavonoids or more flexibly linked dimers, underpinning GK’s notable potency as a multitarget agent [21].

### 2.2. General Pharmacological Activities of Ginkgetin

GK is the first bisflavonoid isolated from *Ginkgo biloba*, from which it derives its name. It is also prevalent in various other medicinal plants. The historical use of these plants in medicine suggests that GK may serve as a fundamental component contributing to their therapeutic effects. For instance, GK is present in *Selaginella* [28], a plant utilized for treating hepatitis and urinary infections, as well as in yew [29], the source of the prominent anticancer drug paclitaxel, and *Taxus* [30,31], which is abundant in antineoplastic alkaloids. Furthermore, plants such as *S. angustifolia* and *S. yunnanensis* also contain GK, which is employed in the treatment of rheumatism, wound infections, and respiratory diseases, respectively [32,33].

Extensive preclinical studies demonstrate that GK exerts a broad range of pharmacological activities across multiple biological systems. GK has been demonstrated to exhibit potent anti-inflammatory and antioxidant effects in preclinical models by suppressing pro-inflammatory signaling pathways and enhancing endogenous antioxidant defense mechanisms [10,16,17]. Moreover, GK displays pronounced anticancer activity by inhibiting tumor cell proliferation, migration and invasion, inducing programmed cell death and suppressing tumor-associated inflammation and angiogenesis [15,20]. In metabolic and cardiovascular disease models, GK improves lipid metabolism, attenuates oxidative stress and preserves tissue structure and function [10,11,18,34]. In neurological disease models, GK confers neuroprotection by mitigating oxidative stress, neuroinflammation and mitochondrial dysfunction [16,19,35]. Additionally, GK exhibits antibacterial and antivirulence activities by interfering with bacterial biofilm formation, quorum sensing and motility, thereby reducing bacterial pathogenicity [36]. Collectively, these pharmacological properties highlight GK as a pleiotropic bioactive compound with considerable therapeutic potential across diverse disease contexts.

## 3. Molecular Mechanisms Underlying the Biological Activities of Ginkgetin

### 3.1. Overview of the Hierarchical Mechanistic Network of Ginkgetin

To fully appreciate the pharmacological profile of GK, it is essential to establish a hierarchical understanding of its multifaceted mechanisms. The current body of evidence reveals that GK operates at multiple levels of biological complexity, ranging from primary molecular interactions to downstream cellular phenotypes.

At the primary level, direct target engagement has been conclusively demonstrated for a limited but expanding set of pathways. Most notably, recent studies utilizing artificial intelligence algorithms combined with biophysical methods have confirmed that GK directly binds to the carboxy-terminal domain of the STING protein, thereby inhibiting STING activation and downstream signal transduction. This direct interaction represents a proximal mechanism that explains GK’s anti-inflammatory and anti-senescence effects [23].

At the secondary level, GK modulates key signaling hubs through indirect mechanisms. For instance, GK activates the Nrf2/HO-1 pathway, as evidenced by increased Nrf2 nuclear translocation and upregulation of antioxidant genes in both in vitro and in vivo models [22]. However, while this association is robust, direct evidence for GK binding to Keap1 (the canonical Nrf2 repressor) to disrupt their interaction is still lacking, positioning Nrf2 activation as a downstream consequence of GK’s primary effects rather than a confirmed direct interaction.

At the tertiary level, these pathway modulations culminate in broad cellular phenotypes, including the regulation of oxidative stress, inflammation, programmed cell death (apoptosis, autophagy, ferroptosis), and tissue remodeling. The following Section 3.2, Section 3.3, Section 3.4, Section 3.5 and Section 3.6 dissect each of these mechanistic layers, with careful attention to distinguishing between well-validated direct targets and associative observations that require further mechanistic validation.

### 3.2. Regulation of Oxidative Stress and Mitochondrial Homeostasis

**Figure 2:**
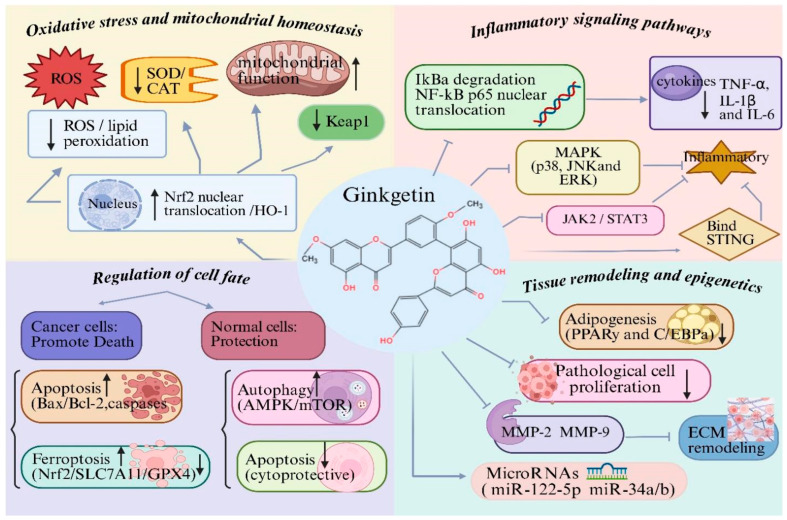
Molecular mechanisms underlying the biological activities of ginkgetin (GK): (1) GK attenuates oxidative stress and maintains mitochondrial homeostasis by reducing ROS and lipid peroxidation, restoring antioxidant enzymes, and activating the Nrf2/HO-1 pathway; (2) GK suppresses inflammatory responses through inhibition of NF-κB, MAPK, JAK2/STAT3 and cGAS–STING signaling, leading to decreased production of pro-inflammatory cytokines (TNF-α, IL-1β and IL-6); (3) GK regulates cell fate in a context-dependent manner by inducing apoptosis or ferroptosis in pathological cells, while protecting normal cells via modulation of autophagy, and suppressing excessive apoptosis; (4) GK inhibits abnormal cell proliferation, adipogenesis and extracellular matrix remodeling, and modulates microRNAs. Created with BioRender. Sun, Z. (2026) https://app.biorender.com/illustrations/696c588a617f6364031a43ca?slideId=f9475d20-5c06-4a34-814e-9b40dd35cef5 (accessed on 20 March 2026).

The cytoprotective effects of GK against oxidative stress are primarily orchestrated by the activation of the Nrf2/HO-1 axis, a central redox-sensitive signaling hub, which subsequently upregulates antioxidant enzymes and preserves mitochondrial function as downstream cytoprotective phenotypes [22]. Oxidative stress is a central pathogenic factor contributing to the initiation and progression of multiple chronic diseases, including metabolic disorders, cardiovascular diseases, neurodegenerative diseases and cancer [37,38,39]. Excessive production of reactive oxygen species (ROS) disrupts redox homeostasis, damages mitochondrial integrity and ultimately leads to cellular dysfunction and death [38,39]. Studies have demonstrated that GK markedly attenuates oxidative stress in diverse experimental models. In metabolic and organ injury models, In vitro studies have demonstrated that GK significantly reduces intracellular ROS levels and lipid peroxidation products in various cell lines under metabolic or inflammatory stress, while animal models of organ injury show that restoring endogenous antioxidant enzyme activities, including superoxide dismutase and catalase [10,17,18]. In parallel, cell-based assays indicate that GK preserves mitochondrial membrane potential, stabilizes mitochondrial function and reduces oxidative stress-induced cellular injury [19,22]. Mechanistically, these antioxidant effects are closely associated with activation of the Nrf2/HO-1 signaling pathway. Both in vitro and in vivo studies consistently show that GK upregulates Nrf2 expression and nuclear translocation while downregulating *Keap1*, thereby enhancing transcription of downstream antioxidant genes and strengthening cellular antioxidant defenses [17,22]. However, it is critical to note that while this association is robust, direct evidence for GK binding to *Keap1* (the canonical Nrf2 repressor) to disrupt their interaction is still lacking. This represents a key knowledge gap, as the observed Nrf2 activation could be an indirect consequence of upstream events rather than a direct molecular interaction. Future studies employing biophysical methods such as surface plasmon resonance (SPR) or cellular thermal shift assay (CETSA) are needed to confirm whether GK directly engages Keap1. Collectively, regulation of oxidative stress and mitochondrial homeostasis constitutes a fundamental mechanism underlying the broad cytoprotective effects of GK (Figure 2). This aligns with the tertiary phenotypic level of the hierarchical framework outlined in Section 3.1.

### 3.3. Modulation of Inflammatory Signaling Pathways

The anti-inflammatory actions of GK stem from its modulation of key inflammatory signaling hubs. Direct mechanistic evidence has recently been provided by studies using artificial intelligence algorithms combined with biophysical methods, which confirmed that GK directly binds to the carboxy-terminal domain of the STING protein, thereby inhibiting STING activation and downstream signal transduction [23]. Chronic inflammation plays a pivotal role in tissue injury [40], fibrosis [41,42] and disease progression across multiple pathological contexts. Beyond this primary target, numerous in vitro and in vivo studies have demonstrated that GK suppresses the NF-κB pathway, as evidenced by inhibition of IκBα degradation and prevention of p65 nuclear translocation, leading to reduced expression of pro-inflammatory cytokines in metabolic, cardiovascular, neurological and musculoskeletal disease models [16,35,43,44]. In addition, cell-based assays and animal models show that GK attenuates MAPK pathway activation, as evidenced by reduced phosphorylation of p38, JNK, and ERK, thereby limiting inflammatory signal amplification [11,35]. It should be noted that while these observations are consistent across multiple models, they represent downstream effects rather than direct inhibition of NF-κB or MAPK components. The modulation of these pathways is likely secondary to GK’s engagement with upstream targets such as STING or other unidentified receptors.

Beyond canonical inflammatory pathways, GK also modulates cytokine-driven inflammation via the JAK/STAT signaling axis. In cancer, hepatic fibrosis and metabolic disease models, both cell-based and animal studies show that GK inhibits phosphorylation of JAK2 and STAT3, resulting in suppression of inflammation-associated cell proliferation, survival and fibrotic responses [15,18,45]. However, direct evidence for GK binding to JAK or STAT proteins is currently absent, and these effects should be interpreted as downstream consequences of primary target engagement. More recently, direct mechanistic evidence has identified GK as a negative regulator of the cGAS-STING pathway through direct binding to STING, thereby inhibiting downstream TBK1, IRF3 and NF-κB activation and alleviating inflammatory and senescence-associated phenotypes [23] (Figure 2). These findings exemplify the primary level of the hierarchical framework outlined in Section 3.1, where direct target engagement (STING binding) leads to modulation of secondary signaling hubs (NF-κB, MAPK) and ultimately culminates in anti-inflammatory phenotypes at the tertiary level.

### 3.4. Regulation of Cell Fate: Apoptosis, Autophagy and Ferroptosis

GK exerts context-dependent control over cell fate decisions by engaging multiple signaling hubs that converge on the core machinery of programmed cell death. In vitro studies have shown that GK modulates the PI3K/Akt/mTOR axis (a secondary pathway) influencing apoptosis and autophagy [10,19]. More recently, direct mechanistic evidence has revealed that GK can activate TFEB, promoting TRIM25-mediated K48-linked polyubiquitination and lysosomal degradation of GPX4, thereby sensitizing cancer cells to ferroptosis [15,46]. Regulation of programmed cell death is a critical determinant of tissue homeostasis and disease outcome. GK has been shown to exert context-dependent regulatory effects on apoptosis, autophagy and ferroptosis.

In malignant cells and activated pathological cells, in vitro studies demonstrate that GK predominantly induces apoptosis through the mitochondrial-dependent pathway. This effect is characterized by an increased *Bax/Bcl-2* ratio, disruption of mitochondrial membrane potential and activation of caspase-9 and caspase-3 [15]. In contrast, in neurons, cardiomyocytes and renal cells exposed to pathological stress, both in vitro and in vivo models show that GK suppresses excessive apoptosis, thereby exerting cytoprotective effects [19,35,43]. This bidirectional regulation highlights the context-dependent nature of GK’s effects, but the underlying molecular switches that determine pro- versus anti-apoptotic outcomes remain poorly understood. In addition, direct mechanistic evidence from cellular assays demonstrates that GK improves experimental colitis by directly targeting and activating EGFR, thereby interfering with PI3K/AKT signaling in intestinal epithelial cells and inhibiting apoptosis [46]. This finding is supported by surface plasmon resonance data showing direct binding of GK to EGFR.

Autophagy represents another critical mechanism underlying GK-mediated protection. In diabetic nephropathy models, in vitro and in vivo studies show that GK restores impaired autophagic flux via activation of the AMPK/mTOR signaling pathway, thereby attenuating oxidative stress, inflammation and extracellular matrix accumulation [10]. In degenerative diseases, such as intervertebral disc degeneration and cerebral ischemia–reperfusion injury, animal models and cell-based assays demonstrate that GK fine-tunes autophagy to prevent excessive autophagic cell death and maintain cellular homeostasis [44,47]. However, whether GK directly activates AMPK or modulates upstream regulators remains unclear, and these effects may be indirect.

Recent studies further reveal that GK participates in the regulation of ferroptosis. In non-small-cell lung cancer, in vitro and in vivo evidence demonstrates that GK synergizes with cisplatin to induce ferroptotic cell death by suppressing the *Nrf2/SLC7A11/GPX4* axis, leading to glutathione depletion and lipid peroxidation [20]. This mechanism expands the spectrum of GK-regulated cell death modalities and suggests potential in overcoming chemoresistance (Figure 2). The regulation of cell fate decisions operates across all three levels of the hierarchical framework: direct target engagement (e.g., EGFR activation, TFEB-mediated GPX4 degradation) at the primary level, modulation of signaling hubs (e.g., PI3K/Akt/mTOR, AMPK) at the secondary level, and the ultimate phenotypic outcomes (apoptosis, autophagy, ferroptosis) at the tertiary level.

### 3.5. Regulation of Cell Proliferation, Differentiation and Tissue Remodeling

The effects of GK on cell proliferation and tissue remodeling are mediated through its impact on key signaling networks such as STAT, PPARγ, and matrix metalloproteinases, as demonstrated by various in vitro and in vivo studies [11,34]. Aberrant cell proliferation, dysregulated differentiation and pathological tissue remodeling are common features of cancer, metabolic disorders and fibrotic diseases [48,49,50]. GK has been shown to modulate these processes through multiple signaling pathways.

In adipose tissue, in vitro differentiation assays and animal models show that GK suppresses adipocyte differentiation by inhibiting STAT5 phosphorylation and downregulating the *PPARy* and *C/EBPa* signaling axis, resulting in reduced lipid accumulation and adipogenesis [11]. While these observations are consistent, direct binding of GK to STAT5 or PPARγ has not been demonstrated, and these effects are likely indirect. In vascular smooth muscle cells, hepatic stellate cells and fibroblasts, cell-based studies and animal models demonstrate that GK interferes with pathological cell proliferation and promotes apoptosis, thereby limiting fibrosis and vascular remodeling [18,43]. However, the molecular targets responsible for these anti-proliferative effects remain to be identified.

GK also plays a significant role in regulating tissue remodeling by modulating extracellular matrix turnover. In vitro and in vivo evidence indicates that GK downregulates matrix metalloproteinases, including MMP-2 and MMP-9, and suppresses excessive extracellular matrix deposition, thereby attenuating pathological remodeling in atherosclerosis, hepatic fibrosis and myocardial infarction models [18,34,43]. It is important to note that these effects on MMPs are likely downstream consequences of GK’s anti-inflammatory and anti-proliferative actions, rather than direct enzyme inhibition, as no evidence for direct MMP binding exists (Figure 2). These observations align with the tertiary (phenotypic) level of the hierarchical framework, representing downstream consequences of primary and secondary pathway modulation.

### 3.6. Epigenetic and Post-Transcriptional Regulation

Emerging evidence suggests that GK can also influence gene expression at the epigenetic and post-transcriptional levels, such as by modulating microRNA expression. These actions, observed primarily in cell-based studies, may represent additional layers of regulation that fine-tune the downstream phenotypic outcomes initiated by primary target engagement [24].

In addition to classical signaling pathways, in vitro evidence suggests that GK exerts regulatory effects at the epigenetic and post-transcriptional levels. GK has been reported to modulate the expression of specific microRNAs, including *miR-122-5p* and *miR-34a/b*, thereby influencing downstream targets involved in apoptosis, proliferation and tumor progression [45]. However, it should be emphasized that these findings are purely associative and derived from correlative expression analyses. No studies have yet demonstrated direct interaction of GK with miRNA processing machinery or epigenetic modifiers. This represents a nascent area of investigation that requires rigorous mechanistic validation, including chromatin immunoprecipitation and miRNA reporter assays, to establish causality (Figure 2). Within the hierarchical framework, these epigenetic effects currently reside at the tertiary phenotypic level, as their association with primary target engagement remains to be established.

## 4. Therapeutic Potential of Ginkgetin Across Disease Systems

### 4.1. Cancer

**Figure 3:**
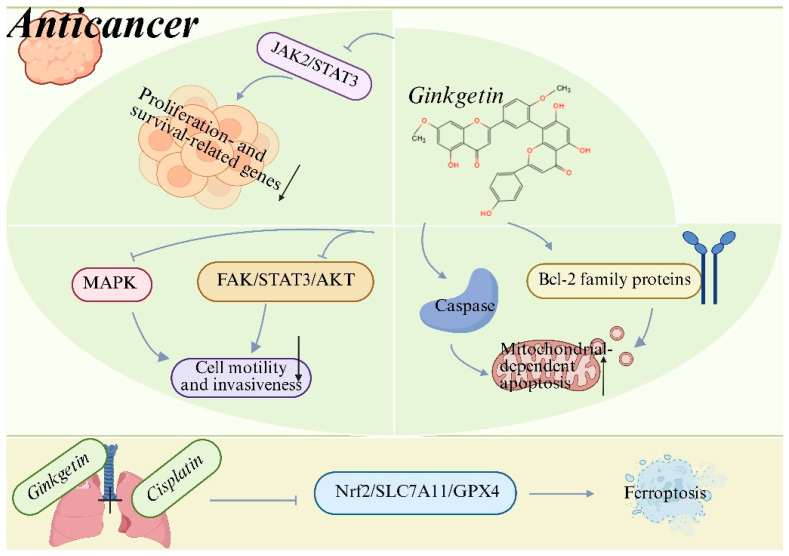
Anticancer mechanisms of ginkgetin (GK). GK suppresses tumor cell proliferation, survival, migration and invasiveness by inhibiting oncogenic signaling pathways, including JAK2/STAT3, MAPK and FAK/STAT3/AKT. Meanwhile, GK triggers mitochondrial-dependent apoptosis through modulation of Bcl-2 family proteins and activation of caspases. Moreover, GK enhances the anticancer efficacy of cisplatin by promoting ferroptosis via suppression of the Nrf2/SLC7A11/GPX4 axis, thereby enhancing chemosensitivity and overcoming drug resistance. Created with BioRender. Sun, Z. (2026) https://app.biorender.com/illustrations/696f4198a93f5e17600f3c3a?slideId=f83d8a3d-b04f-4cfd-8f2c-3d76a99bbc30 (accessed on 20 March 2026).

Preclinical evidence indicates that GK exhibits broad-spectrum anticancer activity across multiple tumor types, including lung cancer [15,20], hepatocellular carcinoma [51], colorectal cancer [52,53], and osteosarcoma [54]. GK effectively suppresses cancer cell proliferation, migration and invasion while inducing programmed cell death in a context-dependent manner. Mechanistically, GK exerts its anticancer effects primarily through inhibition of oncogenic signaling pathways. In multiple cancer models, GK suppresses the JAK2/STAT3 signaling axis, leading to reduced expression of proliferation- and survival-related genes [55,56]. GK also inhibits FAK/STAT3/AKT [20] and MAPK [57] signaling pathways, thereby impairing tumor cell motility and invasiveness. In addition, GK induces mitochondrial-dependent apoptosis characterized by caspase activation and modulation of Bcl-2 family proteins [51,55]. Notably, recent studies demonstrate that GK can synergize with conventional chemotherapeutic agents. In non-small-cell lung cancer, GK enhances cisplatin-induced cytotoxicity by promoting ferroptosis through suppression of the *Nrf2/SLC7A11/GPX4* axis, suggesting a promising strategy to overcome chemoresistance [15]. These findings support the potential of GK as both a standalone and adjuvant anticancer agent (Figure 3) (Table 1). Collectively, these preclinical findings position GK as a candidate worthy of further clinical investigation for cancer. Translation into human therapies, however, is contingent upon future clinical trials that can confirm these effects and establish a favorable safety profile in patients.

#### Critical Appraisal: Translational Challenges and Model Limitations in Oncology

While the preclinical evidence for GK’s anticancer activity is compelling, several critical limitations must be acknowledged when considering its clinical translation. A primary concern is the use of high, often non-physiological concentrations in numerous in vitro studies. Concentrations exceeding 20 μM, while effective in cell-based assays, may not be achievable in human systemic circulation due to its poor aqueous solubility and limited oral bioavailability [9]. Such high doses raise questions about the specificity of observed effects and their relevance to the in vivo situation [24].

Furthermore, while animal tumor models (e.g., xenograft and syngeneic models) have demonstrated GK’s in vivo efficacy [55], they do not fully recapitulate the complexity of human cancers. Factors such as tumor heterogeneity, the intricate tumor microenvironment, and interspecies differences in metabolism and immune function can lead to divergent outcomes between preclinical models and human patients [24]. Additionally, the heterogeneity in dosing regimens, routes of administration, and treatment durations across different animal studies complicates cross-study comparisons and the extrapolation of findings to humans [9].

Despite these limitations, recent studies have made progress in target identification, with STING being confirmed as a direct molecular target of GK [23]. Moving forward, future studies should prioritize the following: (1) using more physiologically relevant concentrations that align with achievable pharmacokinetic profiles; (2) employing advanced model systems such as patient-derived organoids and humanized mouse models [24]; (3) developing optimized formulations (e.g., nano-delivery systems) to enhance bioavailability and tumor targeting [44].

### 4.2. Metabolic Disorders

**Figure 4:**
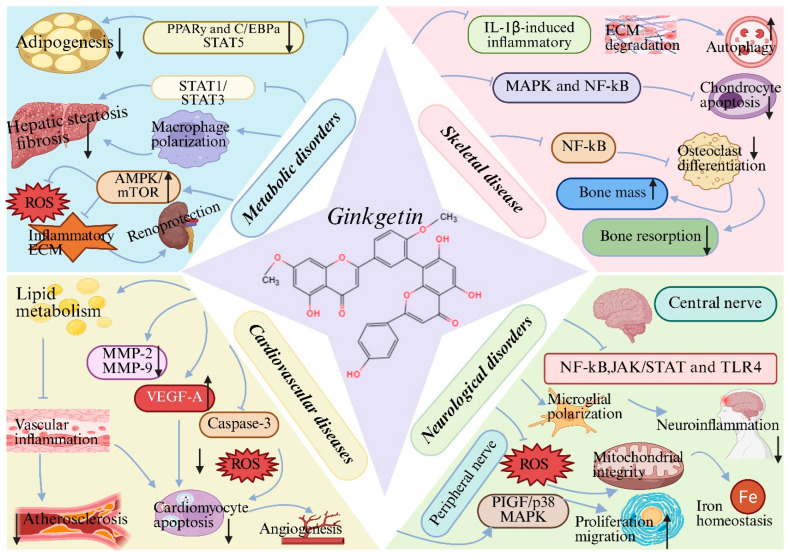
Therapeutic potential of ginkgetin (GK) across disease systems: (1) Metabolic disorders: GK suppresses adipogenesis, hepatic steatosis and fibrosis by inhibiting STAT5 and STAT1/STAT3 signaling, regulating macrophage polarization, and reduces oxidative stress, inflammatory responses and extracellular matrix deposition via the AMPK/mTOR pathway; (2) Cardiovascular diseases: GK improves lipid metabolism, attenuates vascular inflammation and oxidative stress, reduces cardiomyocyte apoptosis, and promotes angiogenesis through modulation of MMP-2/9, VEGF-A and caspase-3; (3) Neurological disorders: GK alleviates neuroinflammation by inhibiting NF-κB, JAK/STAT and TLR4 pathways, preserves mitochondrial integrity and iron homeostasis, and promotes peripheral nerve regeneration via the PlGF/p38 MAPK axis; (4) Skeletal disease: GK suppresses IL-1β-induced inflammation and extracellular matrix degradation, restores autophagic balance, inhibits chondrocyte apoptosis and osteoclast differentiation, reduces bone resorption, and preserves bone mass. Created with BioRender. Sun, Z. (2026) https://app.biorender.com/illustrations/696c580e3c5ff191ef28e1d6?slideId=7bbb521c-814e-457d-9b24-ca82bd9411ee (accessed on 20 March 2026).

Preclinical studies have demonstrated the therapeutic potential of GK in metabolic disorders, including obesity [11], non-alcoholic steatohepatitis (NASH) [45] and diabetic nephropathy [10]. These diseases are characterized by metabolic imbalance, chronic inflammation and oxidative stress, all of which are effectively targeted by GK. In obesity models, GK suppresses adipocyte differentiation and lipid accumulation by inhibiting STAT5 phosphorylation and downregulating the PPARy and C/EBPa signaling cascade, leading to reduced adipogenesis and body weight gain [11]. In NASH models, GK alleviates hepatic steatosis, inflammation and fibrosis by modulating macrophage polarization and suppressing STAT1/STAT3 signaling pathways [18,45]. In diabetic nephropathy, GK exerts renoprotective effects by restoring impaired autophagic flux via activation of the AMPK/mTOR pathway, thereby reducing oxidative stress, inflammatory responses and extracellular matrix deposition [10]. Collectively, these findings indicate that GK acts as a multitarget metabolic regulator with therapeutic potential in complex metabolic diseases (Figure 4) (Table 1). While these preclinical observations are promising, rigorous clinical studies are required to determine whether GK can offer therapeutic benefits for metabolic disorders in human patients.

### 4.3. Cardiovascular Diseases

Preclinical studies have demonstrated that GK exerts cardioprotective effects in various cardiovascular disease models, including atherosclerosis, myocardial infarction and ischemia–reperfusion injury. In atherosclerosis models, GK improves lipid metabolism, suppresses vascular inflammation and inhibits pathological vascular remodeling [34]. In myocardial infarction, GK attenuates oxidative stress, inflammation and apoptosis while promoting angiogenesis and favorable cardiac remodeling. These effects are associated with downregulation of matrix metalloproteinases (MMP-2 and MMP-9) and upregulation of pro-angiogenic factors such as VEGFA [43]. During myocardial ischemia–reperfusion injury, GK significantly reduces infarct size and cardiomyocyte apoptosis by inhibiting caspase-3 activation and inflammatory signaling, thereby preserving cardiac function [58]. Together, these studies highlight the therapeutic promise of GK as an adjunctive agent in cardiovascular disease management (Figure 4) (Table 1). These cardioprotective effects observed in animal models provide a strong rationale for further investigation, although their clinical relevance remains to be validated in human trials.

### 4.4. Neurological Disorders

Preclinical studies suggest that GK possesses broad neuroprotective potential across multiple neurological conditions, primarily mediated through its anti-inflammatory, antioxidant, and anti-apoptotic properties. In Alzheimer’s disease, GK directly targets Aβ pathology by inhibiting β-secretase and modulating fibril formation, while in vivo it reduces Aβ burden and neuroinflammation, partly via NF-κB pathway suppression [13,59,60,61]. Its role in Parkinson’s disease involves mitigating dopaminergic neuron loss; in MPTP-induced models, GK improves motor function, reduces oxidative stress, and inhibits apoptosis. A notable mechanism is its ability to chelate iron and regulate cerebral iron homeostasis, thereby alleviating iron-mediated toxicity [62,63].

In ischemic stroke and cerebral ischemia–reperfusion injury, GK consistently improves neurological outcomes by modulating interconnected pathways. It reduces infarct volume, edema, and oxidative stress while dampening neuroinflammation through inhibition of PI3K/NF-κB/TLR-4 and JAK/STAT signaling [64,65]. Furthermore, it promotes microglial polarization toward the protective M2 phenotype via PPARγ [66] and exerts anti-apoptotic effects by activating the PI3K/Akt/mTOR axis and inhibiting the NF-κB/p53 pathway [19,35,47]. For peripheral nerve repair, GK enhances Schwann cell proliferation and migration via activation of the PIGF/p38 MAPK pathway, facilitating structural and functional recovery [67]. Collectively, these findings position GK as a multitarget agent capable of countering key pathological processes—protein aggregation, oxidative stress, neuroinflammation, and apoptotic/autophagic dysregulation—common to diverse central and peripheral nervous system disorders (Figure 4) (Table 1). Their translation into human therapies for neurological disorders, however, is contingent upon future clinical trials that can confirm these effects and establish a favorable safety profile in patients.

#### Critical Appraisal: Translational Challenges and Model Limitations in Neurology

The neuroprotective effects of GK, while promising, are predominantly derived from preclinical studies that face significant translational hurdles [9]. The issue of achieving therapeutically relevant concentrations in the central nervous system is particularly challenging, as GK must not only reach systemic circulation but also cross the blood–brain barrier (BBB). Its high lipophilicity, while potentially beneficial for BBB penetration, must be balanced against its poor aqueous solubility, and most in vitro neuroprotection studies have employed concentrations that may not reflect achievable brain tissue levels [9].

Additionally, animal models of neurological disorders—such as chemically induced stroke, transgenic Alzheimer’s disease models, and MPTP-induced Parkinson’s disease—recapitulate certain aspects of human pathology but fail to capture the full spectrum of disease heterogeneity, chronic progression, and comorbid conditions characteristic of human patients [24]. The complex interplay between different cell types (neurons, glia, microglia) in the CNS is also difficult to fully replicate in vitro [68].

It is important to note that while *Ginkgo biloba* extracts as a whole have been extensively studied in the context of cognitive function and dementia, these effects cannot be solely attributed to GK due to the presence of multiple bioactive constituents [69]. As of now, there are no large-scale human clinical trials specifically evaluating isolated GK for cognitive or neurological benefits [70]. Future research should focus on the following: (1) rigorous pharmacokinetic studies to determine brain penetration and achievable concentrations; (2) the validation in more sophisticated models, including human iPSC-derived neurons and organoids [71]; (3) the development of targeted delivery systems to enhance CNS exposure [9].

### 4.5. Skeletal Disease

In musculoskeletal diseases, including intervertebral disc degeneration, osteoarthritis and osteoporosis, preclinical studies have shown that GK exerts protective effects by modulating inflammation, oxidative stress and extracellular matrix homeostasis. In intervertebral disc degeneration models, GK suppresses IL-1β-induced inflammatory responses and extracellular matrix degradation in nucleus pulposus cells, while restoring autophagic balance and delaying disc degeneration [44]. In osteoarthritis, GK inhibits MAPK and NF-κB signaling pathways, thereby reducing chondrocyte apoptosis and cartilage destruction [14,35]. Furthermore, GK suppresses osteoclast differentiation and activity by inhibiting NF-κB signaling, leading to reduced bone resorption and preservation of bone mass in osteoporosis models [72,73].These findings support the potential of GK in the management of degenerative musculoskeletal disorders (Figure 4) (Table 1). Nevertheless, these effects have only been demonstrated in preclinical models, and clinical studies are essential to evaluate the therapeutic utility of GK in skeletal diseases.

### 4.6. Antibacterial and Antivirulence Activities

Beyond its effects on host cells, GK exhibits antibacterial and antivirulence properties in vitro and in vivo. Studies have demonstrated its efficacy against Gram-negative *Escherichia coli* by potently inhibiting biofilm formation, a key contributor to chronic infections and antibiotic tolerance. At sub-inhibitory concentrations, GK suppresses the production of extracellular polymeric substances (EPS) and reduces bacterial motility. This anti-biofilm activity is mediated through the downregulation of critical genes involved in curli fiber production (csgA, csgD), flagellar assembly (flhC, flhD, fliC, fliM), and quorum sensing (luxS, lsrB, lsrK, lsrR), thereby disrupting bacterial colonization and community formation [36]. Notably, GK exhibits synergistic effects when combined with antibiotics like gentamicin and colistin against both standard and clinically isolated E. coli strains, highlighting its potential as an adjuvant therapy [36].

Furthermore, GK exhibits anti-virulence properties against the Gram-positive pathogen *Streptococcus suis* serotype 2 (SS2). Its activity is directed against a major virulence factor, suilysin (SLY), a pore-forming toxin responsible for tissue damage and systemic infection. GK dose-dependently inhibits the hemolytic activity of SLY by interfering with its oligomerization process, without affecting bacterial growth or SLY expression [74]. In vitro, GK protects host cells from SS2-induced cytotoxicity by suppressing SLY’s pore-forming activity [74]. In vivo, it reduces bacterial burden in the organs of infected mice [74]. This identifies GK as a potent anti-virulence agent capable of neutralizing a key toxin, thereby mitigating disease severity. These properties expand the therapeutic scope of GK beyond non-infectious diseases (Figure 4) (Table 1). While these findings are encouraging, they are derived from in vitro and animal studies; clinical investigations are needed to determine whether GK’s antibacterial and antivirulence properties can be harnessed for human infectious diseases.

**Table 1:** Summary of Key Preclinical Evidence for Ginkgetin Across Disease Systems.

Disease	Findings	Experimental Models	Mechanisms/Targets	Evidence Level	References
Cancer	Inhibits proliferation, migration, invasion; induces apoptosis and ferroptosis; synergizes with cisplatin	Lung cancer cells (A549, H1975), hepatocellular carcinoma cells (HepG2), colorectal cancer cells (HCT116), osteosarcoma cells (U2OS), xenograft mouse models	Inhibition of JAK2/STAT3, MAPK, FAK/STAT3/AKT; suppression of *Nrf2/SLC7A11/GPX4* axis (ferroptosis); mitochondrial-dependent apoptosis (*Bax/Bcl-2/caspase-3*)	In vitro, in vivo	[15,20,51,55,57]
Metabolic Disorders	Suppresses adipogenesis and lipid accumulation; alleviates hepatic steatosis, inflammation, and fibrosis; restores autophagic flux in diabetic nephropathy	3T3-L1 adipocytes, high-fat diet-induced obese mice, NASH mouse models, high glucose-induced mesangial cells, diabetic nephropathy rat models	Inhibition of *STAT5/PPARγ/C/EBPα*; modulation of macrophage polarization (*STAT1/STAT3*); activation of AMPK/mTOR-mediated autophagy	In vitro, in vivo	[10,11,18,45]
Cardiovascular Diseases	Improves lipid metabolism; attenuates vascular inflammation; reduces infarct size and cardiomyocyte apoptosis; promotes angiogenesis	Atherosclerosis rat models, myocardial infarction mouse models, H9C2 cardiomyocytes under hypoxia/reoxygenation	Downregulation of MMP-2/9; upregulation of VEGFA; inhibition of caspase-3; suppression of inflammatory signaling	In vitro, in vivo	[34,43,58]
Neurological Disorders	Reduces Aβ pathology; protects against dopaminergic neuron loss; improves outcomes in stroke; promotes peripheral nerve repair	APP/PS1 transgenic mice, MPTP-induced Parkinson’s mouse models, cerebral ischemia–reperfusion rat models, Schwann cell	Inhibition of β-secretase, NF-κB, TLR4, JAK/STAT; iron chelation; activation of PI3K/Akt/mTOR, PPARγ; PIGF/p38 MAPK activation	In vitro, in vivo; *direct target* (*iron chelation*)	[35,60,62,66,67]
Skeletal Disease	Suppresses inflammation and ECM degradation in intervertebral disc degeneration; reduces chondrocyte apoptosis in osteoarthritis; inhibits osteoclast differentiation in osteoporosis	IL-1β-treated nucleus pulposus cells, rat disc degeneration models, osteoarthritis rat models, ovariectomized (OVX) mouse models	Inhibition of MAPK (p38, JNK, ERK) and NF-κB signaling; restoration of autophagic balance; suppression of osteoclast differentiation	In vitro, in vivo	[14,35,44,72]
Infectious Diseases	Inhibits biofilm formation; reduces bacterial virulence; synergizes with antibiotics	coli cultures, Streptococcus suis cultures, mouse infection models	Downregulation of biofilm-related genes (*csgA*, *csgD*, *flhC*, *flhD*); inhibition of quorum sensing (*luxS*, *lsrB*); inhibition of suilysin (SLY) oligomerization	*Direct* (*enzyme inhibition*); in vitro, in vivo	[36,74]
Inflammation & Senescence	Alleviates cellular senescence and systemic inflammation; reduces SASP factors	Doxorubicin/IR-induced senescent MEFs, Trex1^−^/^−^ mice, aging mouse models	Direct binding to STING (carboxy-terminal domain); inhibition of STING/TBK1/IRF3/NF-κB signaling	*Direct* (*binding confirmed by SPR/CETSA*)	[23]
Oxidative Stress & Tissue Injury	Reduces ROS and lipid peroxidation; restores antioxidant enzymes; protects against chemical-induced organ injury	Polystyrene microplastics-induced rat liver injury model, H_2_O_2_-treated cell lines	Activation of Nrf2/HO-1 pathway; upregulation of Nrf2 and HO-1; downregulation of *Keap-1*	In vitro, in vivo	[17,22]

## 5. Pharmacokinetic Considerations and Druggability Challenges

### 5.1. Key Pharmacokinetic and Druggability Challenges

Despite the broad pharmacological activities and promising therapeutic potential of GK, several pharmacokinetic and druggability challenges must be carefully considered before its clinical translation. GK exhibits relatively low oral bioavailability due to its biflavonoid structure and high lipophilicity, which result in poor aqueous solubility and limited intestinal absorption [9,10]. Following absorption, GK is susceptible to extensive first-pass metabolism, further reducing systemic exposure [10]. Detailed information regarding its metabolic pathways, active metabolites, and clearance mechanisms remains scarce [9]. Moreover, the tissue distribution profile of GK has not been comprehensively characterized, making it difficult to accurately predict target organ exposure and therapeutic windows across different disease systems [10,18]. The lack of standardized dosing regimens across existing preclinical studies complicates cross-study comparisons and extrapolation to humans [9].Although most studies report no obvious toxicity at pharmacologically effective doses, systematic toxicological evaluations-including long-term administration, reproductive toxicity and organ-specific safety-are still lacking [9]. Furthermore, the intrinsic multi-target nature of GK, while advantageous for pleiotropic effects, raises concerns regarding potential off-target effects and drug–drug interactions, particularly when combined with conventional therapies [15,20].

### 5.2. Innovative Strategies to Overcome Druggability Hurdles

To overcome these pharmacokinetic limitations, several innovative strategies have been proposed and are under active investigation. These approaches aim to enhance GK’s solubility, bioavailability, and target specificity, thereby improving its therapeutic index and facilitate clinical translation.

#### 5.2.1. Nano-Formulation Strategies

Advanced drug delivery systems, particularly nano-formulations, have emerged as a powerful tool to improve the oral bioavailability and targeted delivery of hydrophobic natural products like GK. Liposomes, polymeric nanoparticles, and solid lipid nanoparticles can encapsulate GK, protecting it from premature degradation in the gastrointestinal tract and first-pass metabolism, while also prolonging its circulation time [9]. A recent study developed a conditionally sequential delivery system using an MMP13-responsive nanoplatform for co-delivery of GK and rapamycin, demonstrating enhanced efficacy in intervertebral disc degeneration models. This work exemplifies the potential of nano-carriers to achieve spatiotemporal control over GK release and improve its therapeutic outcome [44]. Furthermore, surface modification of nanoparticles with targeting ligands (e.g., antibodies, peptides) could enable active targeting to specific tissues or cells, such as tumors or inflamed sites, thereby reducing off-target effects [9].

#### 5.2.2. Prodrug Design

Prodrug approaches involve the chemical modification of GK to temporarily mask its polar or lipophilic groups, thereby enhancing its aqueous solubility or membrane permeability. Upon absorption, the prodrug is enzymatically or chemically converted to the active parent compound in vivo. This strategy has been successfully applied to many polyphenolic compounds and could be tailored to GK’s structure to improve its oral bioavailability [9]. For example, phosphorylation or glycosylation of hydroxyl groups could increase water solubility, while esterification of hydroxyls with lipophilic moieties might enhance membrane transport [9].

#### 5.2.3. Co-Delivery and Combination Approaches

The co-administration or co-formulation of GK with other agents that modulate its pharmacokinetics or pharmacodynamics represents another promising avenue. Combining GK with absorption enhancers (e.g., piperine) could inhibit intestinal and hepatic metabolism, thereby increasing systemic exposure [9]. Additionally, the synergistic effects of GK with conventional chemotherapeutics, as demonstrated in cancer models (e.g., with cisplatin [20], suggest that combination therapy might allow dose reduction in both agents, mitigating toxicity while maintaining efficacy. Such combination strategies could also be integrated with nano-formulations to achieve co-delivery of multiple agents [44].

#### 5.2.4. Structural Modification Guided by SAR

Insights from SAR studies provide a rational basis for designing GK analogs with improved drug-like properties. By identifying the functional groups essential for target engagement (e.g., STING binding) versus those contributing primarily to lipophilicity, medicinal chemistry efforts could generate simplified analogs or novel biflavonoids with enhanced solubility, metabolic stability, and target selectivity [9]. However, such modifications must be carefully balanced to preserve the multi-target pharmacological profile that underpins GK’s therapeutic potential.

In summary, a multifaceted approach combining advanced nano-formulations, prodrug design, co-delivery strategies, and SAR-driven structural optimization holds great promise to overcome GK’s pharmacokinetic limitations. Continued research in these areas is essential to unlock its full therapeutic potential.

## 6. Limitations and Future Perspectives

Despite the compelling pharmacological activities of GK demonstrated in diverse disease models, several limitations should be acknowledged. First, as critically appraised in Sections “Critical Appraisal: Translational Challenges and Model Limitations in Oncology” and “Critical Appraisal: Translational Challenges and Model Limitations in Neurology”, the majority of current evidence is derived from in vitro and animal studies, with a complete absence of clinical data. Differences in disease models, dosing regimens and treatment durations across studies further complicate direct comparisons and translational interpretation. Second, the pharmacokinetic properties of GK remain insufficiently characterized Issues such as low oral bioavailability, limited tissue distribution, and unclear metabolic pathways may restrict its clinical applicability. Comprehensive pharmacokinetic and toxicological studies are therefore required to optimize dosing strategies and ensure long-term safety. Third, although GK modulates multiple signaling pathways, the direct molecular targets responsible for its pleiotropic effects remain incompletely defined. Future studies employing target identification approaches, such as proteomics, chemical biology and genetic validation, will be essential to delineate its primary targets and off-target effects. From a translational perspective, future research should prioritize the development of optimized formulations, including structural modification and nanodelivery improve bioavailability and tissue targeting. In addition, the synergistic potential of GK with existing therapeutic agents warrants systematic investigation, particularly in cancer, metabolic and inflammatory diseases. In conclusion, GK represents a promising multitarget natural compound with broad therapeutic potential. Addressing the current limitations through rigorous mechanistic, pharmacokinetic and clinical studies will be critical for advancing GK from bench to bedside.

## 7. Conclusions and a Translational Roadmap

GK, a naturally occurring biflavonoid primarily isolated from *Ginkgo biloba*, has emerged as a multifaceted bioactive compound with significant therapeutic promise across a broad spectrum of diseases. As extensively discussed, its efficacy stems from its ability to coordinately modulate a complex network of key signaling path-ways—including Nrf2/HO-1, NF-κB, JAK/STAT, MAPKs, PI3K/Akt/mTOR, and cGAS-STING—that are critically involved in oxidative stress, inflammation, cell death, and tissue remodeling. This multitarget mechanism underpins GK’s demonstrated protective effects in preclinical models of cancer, metabolic disorders, cardiovascular and neurological diseases, skeletal disorders, and bacterial infections.

Although GK represents a highly promising preclinical lead compound, the most immediate and critical step forward is the design and execution of well-controlled clinical trials to evaluate whether this significant preclinical promise translates into tangible therapeutic benefits for patients. Addressing existing pharmacokinetic and safety challenges will be essential for this transition from bench to bedside.

To systematically advance GK from preclinical promise to clinical candidacy, a focused five-year roadmap is essential. The immediate priority (Years 1–2) must be definitive target validation (e.g., using biophysical assays to confirm direct binding to STING and Keap1) and the selection of an optimized formulation (e.g., prodrug or nanocarrier) that achieves pharmacologically relevant systemic exposure. Subsequently (Years 2–4), efforts should shift to comprehensive preclinical de-risking, including GLP-compliant toxicology studies, PK/PD modeling, and CMC development for the lead candidate. Finally (Years 4–5+), clinical translation should be initiated with a Phase 0 microdosing study to confirm human pharmacokinetics, followed by a Phase I trial to establish safety and a recommended Phase 2 dose. This structured pathway, centered on solving the key challenges of target verification, bioavailability, and safety, provides a clear and actionable strategy for harnessing the full clinical potential of GK.

## Data Availability

Not applicable.

## Abbreviations

GK—Ginkgetin; ROS—Reactive oxygen species; Nrf2—Nuclear factor erythroid 2–related factor 2; HO-1—Heme oxygenase-1; NF-κB—Nuclear factor kappa-B; JAK—Janus kinase; STAT—Signal transducer and activator of transcription; MAPKs—Mitogen-activated protein kinases; AMPK—AMP-activated protein kinase; mTOR—Mechanistic target of rapamycin; PI3K—Phosphoinositide 3-kinase; Akt—Protein kinase B; cGAS—Cyclic GMP-AMP synthase; STING—Stimulator of interferon genes; TBK1—TANK-binding kinase 1; IRF3—Interferon regulatory factor 3; TNF-α—Tumor necrosis factor-alpha; IL—Interleukin; MMP—Matrix metalloproteinase; VEGFA—Vascular endothelial growth factor A; PPARγ—Peroxisome proliferator-activated receptor gamma; C/EBPα—CCAAT/enhancer-binding protein alpha; NASH—Non-alcoholic steatohepatitis; TLR4—Toll-like receptor 4; EPS—Extracellular polymeric substances; SLY—Suilysin; SS2—*Streptococcus suis* serotype 2.
